# Impact of point‐of‐care HIV viral load and targeted drug resistance mutation testing on viral suppression among Kenyan pregnant and postpartum women: results from a prospective cohort study (Opt4Mamas)

**DOI:** 10.1002/jia2.26182

**Published:** 2023-11-08

**Authors:** Rena C. Patel, Patrick Oyaro, Katherine K. Thomas, Garoma Wakjira Basha, James Wagude, Irene Mukui, Evelyn Brown, Shukri A. Hassan, Eunice Kinywa, Fredrick Oluoch, Francesca Odhiambo, Boaz Oyaro, Leonard Kingwara, Enericah Karauki, Nashon Yongo, Lindah Otieno, Grace C. John‐Stewart, Lisa L. Abuogi

**Affiliations:** ^1^ Department of Medicine University of Washington Seattle Washington USA; ^2^ Department of Global Health University of Washington Seattle Washington USA; ^3^ Department of Medicine University of Alabama Birmingham UK; ^4^ Health Innovations Kenya (HIK) Kisumu Kenya; ^5^ Department of Health Siaya County Kenya; ^6^ Drugs for Neglected Diseases Initiative (DNDI) Nairobi Kenya; ^7^ UWKenya Nairobi Kenya; ^8^ Department of Health Kisumu County Kenya; ^9^ Family AIDS Care and Education Services Kenya Medical Research Institute Kisumu Kenya; ^10^ Kenya Medical Research Institute‐CDC Kisian Kenya; ^11^ National HIV Reference Laboratory Kenya Ministry of Health Nairobi Kenya; ^12^ Departments of Pediatrics and Epidemiology University of Washington Seattle Washington USA; ^13^ Department of Pediatrics University of Colorado Denver Colorado USA

**Keywords:** HIV, pregnant and postpartum women, antiretroviral therapy (ART), point‐of‐care (POC) testing, viral load, drug resistance mutations (DRMs)

## Abstract

**Introduction:**

Lack of viral suppression (VS) among pregnant and breastfeeding women living with HIV poses challenges for maternal and infant health, and viral load (VL) monitoring via centralized laboratory systems faces many barriers. We aimed to determine the impact of point‐of‐care (POC) VL and targeted drug resistance mutation (DRM) testing in improving VS among pregnant and postpartum women on antiretroviral therapy.

**Methods:**

We conducted a pre/post‐intervention prospective cohort study among 820 pregnant women accessing HIV care at five public‐sector facilities in western Kenya from 2019 to 2022. The pre‐intervention or “control” group consisted of standard‐of‐care (SOC) centralized VL testing every 6 months and the post‐intervention or “intervention” group consisted of a combined strategy of POC VL every 3 months, targeted DRM testing, and clinical management support. The primary outcome was VS (VL ≤1000 copies/ml) at 6 months postpartum; secondary outcomes included uptake and turnaround times for VL testing and sustained VS.

**Results:**

At 6 months postpartum, 321/328 (98%) of participants in the intervention group and 339/347 (98%) in the control group achieved VS (aRR 1.00, 95% confidence interval [CI] 0.98, 1.02). When assessing VS using a threshold of <40 copies/ml, VS proportions were lower overall (90−91%) but remained similar between groups. Among women with viraemia (VL>1000 copies/ml) who underwent successful DRM testing in the intervention group, all (46/46, 100%) had some DRMs and 20 (43%) had major DRMs (of which 80% were nucleos(t)ide reverse transcriptase inhibitor mutations). POC VL testing uptake was high (>89%) throughout pregnancy, delivery, and postpartum periods, with a median turnaround time of 1 day (IQR 1, 4) for POC VL in the intervention group and 7 days (IQR 5, 9) for SOC VL in the control group. Sustained VS throughout follow‐up was similar between groups with either POC or SOC VL testing (90−91% for <1000 copies/ml, 62–70% for <40 copies/ml).

**Conclusions:**

Our combined strategy markedly decreased turnaround time but did not increase VS rates, which were already very high, or sustained VS among pregnant and postpartum women living with HIV. Further research on how best to utilize POC VL and DRM testing is needed to optimize sustained VS among this population.

## 1 INTRODUCTION

Globally, an estimated 1.3 million pregnant women were living with HIV in 2021 [1]. Given the widespread scale‐up of universal antiretroviral therapy (ART) initiation for all individuals, an estimated 70–95% of pregnant women are on ART [2]. However, women do not always achieve or maintain viral suppression (VS), with 22–30% of women having at least one episode of viral load (VL) >1000 copies/ml during pregnancy or postpartum periods [3, 4]. Maternal HIV VL is the leading determinant of vertical transmission of HIV [5, 6], and lack of VS during critical periods of pregnancy and breastfeeding poses serious challenges to eliminating vertical transmission.

Routine VL monitoring while on ART is recommended in low‐ and middle‐income countries (LMICs) [7], but implementation is incomplete. In Kenya, vertical transmission rates decreased from 11.5% in 2017 to 8.9% in 2020, but the country remains short of its target of <5% [8, 9]. Current Kenya guidelines recommend VL monitoring at the first antenatal care (ANC) visit if already on ART or at 6 months post‐ART initiation for newly diagnosed women, and every 6 months postpartum while breastfeeding [10]. While estimates for repeat testing are largely lacking, 86% of all estimated people on ART have undergone at least one VL test in Kenya [11, 12]. VL testing occurs via centralized laboratory testing, which includes several challenges, such as long turnaround times and high costs of transporting samples [10, 13]. Point‐of‐care (POC), or even near POC, VL assessments have been shown to be feasible, accurate, and less expensive than laboratory‐based VL assays [14−18]. Kenya has a nationwide POC tuberculosis testing platform using GeneXpert technology which has been used to pilot HIV early infant diagnosis and VL testing [19−21]. However, the clinical impact of POC VL has been mixed [22−26], and the feasibility of its use during the dynamic periods of pregnancy and postpartum remains unclear.

While improved HIV VL monitoring would enhance the detection of viraemia (VL>1000 copies/ml), a variety of underlying causes exist for the lack of VS among pregnant and postpartum women, including HIV drug resistance mutations (DRMs) [27]. According to the 2021 WHO report on HIV DRMs, non‐nucleos(t)ide reverse transcriptase inhibitors (NNRTIs) resistance has reached a critical level (>10%) in five African countries [28]. With increasing rates of DRMs in LMICs, HIV DRMs could jeopardize the attainment of the global targets for HIV among pregnant women, which have additional implications for transmitted resistance to infants [7]. Nearly half of HIV‐infected infants have transmitted DRMs to one or more NNRTIs [29]. Current challenges in DRM monitoring in Kenya include pre‐consultation with centralized committees, extremely delayed turn‐around times, and testing only for those failing second‐ or third‐line regimens [15, 30−32]. Incorporating DRM testing into clinical decision‐making in LMICs has increased saliency, yet many questions remain on how to implement this testing programmatically and for which priority populations [33, 34].

We conducted the Opt4Mamas study to evaluate if a combined strategy of higher frequency POC VL with targeted DRM testing and clinical decision support could improve VS rates among pregnant and postpartum women on ART in Kenya. We hypothesized that our combined strategy would facilitate earlier and more appropriate clinical decision‐making, resulting in improved treatment outcomes.

## 2 METHODS

### 2.1 Study design and procedures

**Figure 1:**
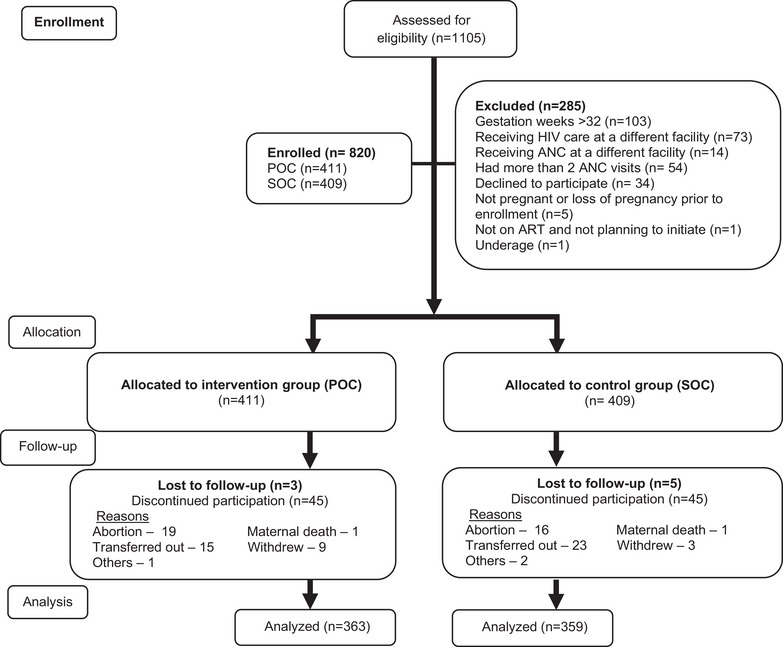
Flow diagram of study participants in the Opt4Mamas study, February 2019–December 2021.

We conducted an open‐label, pre/post‐intervention (or intervention/control) prospective cohort study, enrolling pregnant women living with HIV during their ANC care and followed them through 6 months postpartum in five public‐sector HIV treatment facilities in Kenya from February 2019 to November 2022 (Figure 1). The pre‐intervention cohort served as the “control” group, receiving standard‐of‐care (SOC), which consisted of centralized VL testing approximately every 6 months, from all five facilities. The post‐intervention cohort served as the “intervention” group, receiving a combined strategy of POC VL every 3 months, targeted DRM testing, and clinical management support, also from all five facilities. HIV DRM testing was performed at the two accredited centralized laboratories in Kenya using Sanger sequencing for participants in the intervention group with VL ≥ 1000 copies/ml. Follow‐up of the control cohort overlapped in calendar time with enrolment of the intervention cohort (control group enrolment started on 26 February 2019 and follow‐up lasted until 22 April 2021; intervention group enrolment started on 7 October 2019 and follow‐up lasted until 31 December 2021). We chose the study facilities to leverage existing POC technologies, specifically the GeneXpert platform, and for geographical reach for study staff based in Kisumu, Kenya. Details on study procedures are found in Supplementary Text and Figure 1.

### 2.2 Study setting and population

The study was conducted in low‐resource, high‐HIV burden public sector facilities in Kisumu County, western Kenya, with one of the highest prevalence of HIV infections in Kenya (Table S1). Comprehensive HIV care and treatment services were provided per Kenyan ART guidelines by facility staff, including ART for all persons diagnosed with HIV [10]. First‐line ART regimens among adults during the study period included combinations of two nucleos(t)ide reverse transcriptase inhibitors (NRTIs) lamivudine and tenofovir with either (1) NNRTI efavirenz or (2) integrase inhibitors (INSTIs) dolutegravir, and protease inhibitors (PIs)‐containing ART, for example with atazanavir/ritonavir, were considered alternative first‐line or second‐line regimens; a wider transition for patients on non‐dolutegravir first‐ or second‐line regimens to dolutegravir‐containing ART occurred in 2019 [10].

### 2.3 Study eligibility

We enrolled women presenting at ANC meeting the following inclusion criteria: (1) adult (>17 years of age) pregnant female either already known or newly diagnosed with HIV; (2) at 32 weeks or less of gestational age; (3) with first ANC visit; (4) planning to return to the same facility for the remainder of her pregnancy and postpartum care; and (5) on ART or planning to initiate ART within 1 week. Women on third or salvage ART regimens (due to the higher complexity of managing viraemia [VL>1000 copies/ml] for such individuals) or receiving ANC or HIV care at another facility were excluded.

### 2.4 Variables

Our primary outcome was VS (defined as VL <1000 copies/ml, as per country guidelines) by POC VL testing at 6 months postpartum (defined as 48 weeks +/− 16 weeks, a wide window intended to maximize our ability to obtain a VS measurement in spite of COVID interruptions). If a POC VL test was not available at 6 months postpartum, any available SOC test in the same window was used. Our secondary outcomes included VS defined by lower VL cutoffs and a set of process outcomes, such as uptake and turnaround time of VL testing results. We define major, minor, and accessory classifications for HIV drug resistance according to the Stanford HIV Resistance Database [35]. We define sustained VS (SVS) as having VS at all intervals through the 6‐month postpartum visit among participants with at least two study intervals tested and with the respective VL testing modality for the group (i.e. POC VL for intervention or SOC VL for control group).

The primary exposure was control versus intervention group. Participant clinical and socio‐demographic information as well as household characteristics (e.g. household commodities and food insecurity) were collected from all participants at enrolment. Other information, such as psychosocial and behavioural data (e.g. self‐reported adherence), were collected at every study visit.

### 2.5 Statistical analysis

Power for the study was for comparing the proportion of women with VS 6 months postpartum in the control versus intervention groups. Based on historical facility data, we expected approximately 75% VS in the control group. We estimated that 270 women per group would provide 80% power to detect an increase of 10% post‐intervention VS (i.e. 75% vs. 85%) using a chi‐squared test with α = 0.05. We aimed to screen 350 women in each group to account for an anticipated 25% of available women not enrolling, transferring out to other facilities, experiencing pregnancy loss or lost to follow‐up at 6 months postpartum. In February 2020, we generated additional power calculations given higher‐than‐expected baseline VS. We estimated that with 410 women per group and 90.5% VS in the control group, we would have approximately 80% power to detect a 50% decrease in the proportion unsuppressed (i.e. 9.5% vs. 4.7% unsuppressed).

We compared descriptive statistics for participant baseline characteristics by group using chi‐squared tests for categorical and *t*‐tests for continuous variables. We describe VS by group at enrolment (any blood draw 0–90 days prior to enrolment), 3 and 6 months after enrolment, delivery, and 3 and 6 months postpartum (any blood draw +/− 6 weeks within visit target except for the 3‐month visit which additionally included blood draws from day after enrolment to 6 weeks after the 3‐month visit). The primary analysis compared the observed proportion of women with VS at 6 months postpartum (primary outcome) in the control versus intervention groups using a modified Poisson regression model with robust standard error estimation, adjusting for facility, to obtain the adjusted relative risk (aRR) [36]. An *a priori* sensitivity analysis defined VS as VL<40 copies/ml and *a post hoc* analysis as VL<400 copies/ml. Further *post hoc* sensitivity analyses included: (a) adjusting for enrolment characteristics differing (α<0.05) by group, in case of confounding; and (b) using inverse probability weighting, to address missing outcomes. We also conducted a *post hoc* secondary analysis evaluating the effect of the intervention on SVS.

## 3 RESULTS

### 3.1 Enrolment characteristics

A total of 1105 women were assessed for study eligibility, of which 285 (26%) were excluded (Figure 1). Eight hundred and twenty women were enrolled, with 411 and 409 allocated to the intervention and control groups, respectively. Retention to study visit at 6 months postpartum was 88% and 92% and primary outcome ascertained for 80% and 85% of participants in the intervention and control groups, respectively, and two maternal deaths occurred unrelated to the study.

Median maternal age at enrolment was 29 years (interquartile range [IQR] 24, 33), gestational age was 19 weeks (IQR 13, 25), gravida was 3 (IQR 2, 4), parity was 2 (IQR 1, 3), 423 (52%) had achieved a secondary education and 696 (85%) were married (Table 1). The median CD4 cell count was 523 (IQR 370, 708.5) and 628 (76.6%) were WHO Stage I or II. Overall, 622 (76%) participants were on NNRTI‐, 71 (8.7%) on PI‐ and 80 (10%) on integrase‐containing ART at enrolment, and the median time on ART was 4.0 years (IQR 1.2, 6.3). Among 771 (94%) of the participants with a VL documented in the 24 months prior to enrolment, 672 (82%) had VS.

**Table 1:** Characteristics at enrolment for enrolled participants in the Opt4Mamas study, February 2019–December 2021

Variable	Total study group (*n*=820)	Intervention group (POC VL; *n*=411)	Control group (SOC VL; *n*=409)	*p*‐Value^a^
*Participant characteristics*				
Maternal age (median, IQR)	29 (24, 33)	29 (24, 33)	29 (24, 33)	0.641
Gestational age in weeks (median, IQR)	19 (13, 25)	18 (14, 24)	20 (12, 26)	0.1886
Gravida (i.e. number of pregnancies)	3 (2, 4)	3 (2, 4)	3 (2, 4)	0.9447
Parity (i.e. number of live births)	2 (1, 3)	2 (1, 3)	2 (1, 3)	0.9045
Age at ART initiation in years (median, IQR)	24 (21, 28)	24 (21, 28)	24 (21, 28)	0.1053
Time on ART in years (median, IQR)	3 (1, 6)	4 (1, 6)	3 (1, 6)	0.5614
ART regimen *n* (%)				<0.0001
NNRTI‐containing	622 (75.9%)	272 (66.2%)	350 (85.6%)	
PI‐containing	71 (8.7%)	39 (9.5%)	32 (7.8%)	
Integrase‐containing	80 (9.8%)	70 (17.0%)	10 (2.4%)	
Missing	47 (5.7%)	30 (7.3%)	17 (4.2%)	
CD4 count, most recent recorded within prior 2 years or on day of enrolment *n* (%)				0.8492
0−200	14 (1.7%)	6 (1.5%)	8 (2.0%)	
201−500	82 (10.0%)	29 (7.1%)	53 (13.0%)	
501+	107 (13.0%)	38 (9.3%)	69 (17.0%)	
Missing	617 (75.2%)	338 (82.2%)	279 (68.2%)	
Median CD4, IQR	523 (370, 708.5)	523 (366, 719)	523 (381.3, 706)	0.9484
WHO Clinical Stage, most recent recorded within prior 2 years or on day of enrolment *n* (%)				0.289
I or II	628 (76.6%)	266 (64.7%)	362 (88.5%)	
III or IV	64 (7.8%)	32(8.0%)	32 (7.8%)	
Not indicated or missing	128 (15.6%)	113 (27.5%)	15 (3.7%)	
Viral suppression (<1000 copies/ml via SOC), as closest VL prior to 2 years to or on day of enrolment *n* (%)				0.4918
Yes	672 (82.0%)	326 (79.3%)	346 (84.6%)	
No	99 (12.0%)	41 (10.0%)	58 (14.2%)	
Missing	49 (6.0%)	44 (10.7%)	5 (1.2%)	
***Socio‐demographic characteristics***				
Highest education attained *n* (%)				0.9103
No education	14 (1.7%)	8 (2.0%)	6 (1.5%)	
Primary	390 (47.6%)	196 (47.7%)	194 (47.4%)	
Secondary	303 (37.0%)	153 (37.2%)	150 (36.7%)	
Higher	113 (13.8%)	54 (13.1%)	59 (14.4%)	
Marital status *n* (%)^b^				0.7685
Married	696 (84.9%)	346 (84.2%)	350 (85.6%)	
Not married	122 (14.9%)	63 (15.3%)	59 (14.4%)	
Missing	2 (0.2%)	2 (0.5%)	0	
***Household or partner characteristics***				
HIV status of primary sexual partner *n* (%)				0.652
Positive	442 (53.9%)	222 (54.0%)	220 (53.8%)	
Negative	238 (29.0%)	123 (29.9%)	115 (28.1%)	
Unknown	139 (17.0%)	65 (15.8%)	74 (18.1%)	
Missing	1 (0.1%)	1 (0.2%)	0	
Having household commodities *n* (%)				
Electricity	528 (64.4%)	270 (65.7%)	258 (63.1%)	0.5653
Radio	641 (78.1%)	313 (76.2%)	328 (80.2%)	0.2793
Television	457 (55.7%)	224 (54.5%)	233 (57.0%)	0.8051
Phone	757 (92.3%)	369 (89.8%)	388 (94.9%)	0.0120
Kind of floor^c^	555 (67.7%)	277 (67.7%)	278 (68.1%)	0.9403
More than one room	580 (70.7%)	274 (66.7%)	306 (74.8%)	0.0204
Firewood/plant waste	423 (51.6%)	223 (54.3%)	200 (48.9%)	0.2808
Reporting food insecurity *n* (%)				0.0514
None 0	284 (34.6%)	158 (38.4%)	126 (30.8%)	
Mild 1−9	355 (43.3%)	159 (38.7%)	196 (47.9%)	
Moderate 10−18	169 (20.6%)	87 (21.2%)	82 (20.0%)	
Severe 19−27	11 (1.3%)	6 (1.5%)	5 (1.2%)	
Missing	1 (0.1%)	1 (0.2%)		

Abbreviations: ART, antiretroviral therapy; IQR, inter quartile range; POC, point‐of‐care; SOC, standard‐of‐care; VL, viral load.

^a^ *p*‐Values estimated by Fisher's exact test for categorical variables and Wilcoxon test for continuous variables.

^b^ Various marital status categories include married and cohabiting, married but not co‐habitating, not married but co‐habitating. Not married status categories include single, widowed, separated or divorced.

^c^ Primary kind of floor of the main house can be carpet, cement, tile, earth/dung or others.

More women in the intervention group versus control group were on INSTI‐containing ART (17% vs. 2.4%, respectively, *p*<0.001) and reported no food insecurity (38% vs. 31%, respectively, *p* = 0.05). Facility characteristics, ART regimen changes, VS during the course of the study and delivery outcomes can be found in Tables S1–S4, respectively.

The proportion of women with VS at enrolment in the intervention group was 343/379 (90%) by POC VL testing, and 256/269 (95%) in the control group by SOC VL testing (Table 2). Among those in the intervention group who also had SOC VL testing, VS was 152/157 (97%).

**Table 2:** Effect of the intervention on viral suppression proportions by time and varying threshold of VL cutoffs among the Opt4Mamas study participants (*n*=820), February 2019−December 2021

			Unadjusted model^a^		Adjusted model^b^		IPW model^c^	
	Intervention group (*n*=411)	Control group (*n*=409)	Risk ratio (RR, 95% CI) Risk difference (RD, 95% CI)	*p*‐value for each	Risk ratio (RR, 95% CI) Risk difference (RD, 95% CI)	*p*‐value for each	Risk ratio (RR, 95% CI) Risk difference (RD, 95% CI)	*p*‐value for each
***Viral suppression <1000 copies/ml and baseline subgroups***								
***Viral suppression <1000 copies/ml by testing interval*** ^d^								
Enrolment	343/379 (90.2%)	256/269 (95.2%)	−	−				
3 months	95/98 (97.0%)	103/108 (95.4%)	−	−				
6 months	21/22 (95.5%)	60/60 (100%)	−	−				
Delivery	218/225 (96.9%)	127/133 (95.5%)	−	−				
3 months postpartum	182/186 (97.8%)	144/150 (96.0%)	−	−				
6 months postpartum	208/212 (98.1%)	169/176 (96.0%)	−	−				
**Primary outcome** ^e^ **(6 months postpartum +/− 16 weeks)**	**321/328 (97.9%)**	**339/347 (97.7%)**	**Primary analysis**: **RR: 1.04 (0.36, 3.02)** **RD: 0.2% (−.2.1%, 2.4%)**	**0.941** **0.880**	**RR: 1.0 (0.98, 1.03)** **RD: 0.2% (−.2.1%, 2.4%)**	**0.962** **0.880**	**RR: 1.00 (0.98, 1.02)** **RD: −0.05% (−2.1%, 2.0%)**	**0.653** **0.962**
***Baseline subgroups***								
ART regimen								
NNRTI‐containing	202/206 (98.1%)	293/296 (99.0%)			RR: 1.00 RD: −1.0% (−3.1%, 1.3%)	0.409 0.020	RR: 1.00 RD: −0.8% (−2.8%, 1.2%)	0.425 0.022
PI‐containing	33/35 (94.3%)	25/30 (83.3%)			RR: 0.86 (0.76, 0.98) RD: 11.0% (−4.4%, 26.3%)	0.163 0.745	RR: 0.90 (0.83, 0.99) RD: 9.7% (−4.7%, 24.0%)	0.186 0.839
Integrase‐containing	62/63 (98.4%)	7/7 (100%)			RR: 0.99 (0.95, 1.04) RD: −1.6% (−4.7%, 1.5%)	0.313	RR: 1.00 (0.97, 1.03) RD: −1.3% (−3.9%, 1.2%)	0.314
Food insecurity								
None	123/124 (99.0%)	100/101 (99.0%)			RR: 1.00 RD: 0.2% (−2.3%, 2.7%)	0.885	RR: 1.00 RD: 0.2% (−2.0%, 2.3%)	0.863
Mild	121/125 (97.0%)	169/172 (98.0%)			RR: 0.99 (0.96, 1.02) RD: −1.5% (−5.1%, 2.2%)	0.389 0.435	RR: 0.99 (0.97, 1.01) RD: −1.3% (−4.6%, 2.0%)	0.185 0.447
Moderate	72/74 (97.0%)	67/70 (95.7%)			RR: 0.97 (0.93, 1.01 RD: 1.6 (−4.4%, 7.6%)	0.142 0.606	RR: 0.98 (0.95, 1.01) RD: 2.6% (−2.9%, 8.0%)	0.215 0.357
Severe	5/5 (100%)	3/4 (75.0%)			RR: 0.86 (0.63, 1.17) RD: 25% (−17.4%, 67.4%)	0.323 0.248	RR: 0.90 (0.73, 1.10) RD: 25.0% (−17.3%, 67.4%)	0.282 0.248
***Viral suppression <400 copies/ml by testing interval*** ^d^								
Enrolment	333/379 (87.9%)	251/269 (93.3%)	−	−				
3 months	95/98 (97.0%)	102/108 (94.4%)	−	−				
6 months	20/22 (91.0%)	59/60 (98.3%)	−	−				
Delivery	217/225 (96.4%)	124/133 (93.2%)	−	−				
3 months postpartum	181/186 (97.3%)	142/150 (94.7%)	−	−				
6 months postpartum	208/212 (98.1%)	167/175 (95.4%)	−	−				
**Parallel to primary** ^e^ (6 months postpartum +/− 16 weeks)	321/328 (97.9%)	339/347 (97.7%)	RR: 1.04 (0.36,3.02) RD: 0.2% (−.2.1%, 2.4%)	0.9410.880	RR: 1. 00 (0.96,1.04) RD: 0.2% (−.2.1%, 2.4%)	0.962 0.880	RR: 1.00 (0.98, 1.03) RD: −0.05% (−2.1%, 2.0%)	0.835 0.987
***Viral suppression <40 copies/ml by testing interval*** ^d^								
Enrolment	294/379 (77.6%)	210/269 (71.1%)						
3 months after enrolment	82/98 (83.7%)	78/108 (72.2%)	−	−				
6 months after enrolment	18/22 (81.8%)	43/60 (71.7%)	−	−				
Delivery	198/225 (88.0%)	95/133 (71.4%)	−	−				
3 months postpartum	166/186 (89.2%)	115/150 (76.7%)	−	−				
6 months postpartum	189/212 (89.2%)	144/176 (81.8%)	−	−				
**Parallel to primary** ^e^ (6 months postpartum +/− 16 weeks)	297/328 (90.5%)	311/347 (89.6%)	RR: 1.01 (0.59,1.71) RD: 1.0% (−3.6%, 5.4%)	0.976 0.688	RR: 1.01 (0.96,1.10) RD: 1.0% (−3.6%, 5.4%)	0.732 0.688	RR: 1.01 (0.95, 1.06) RD: 0.5% (−3.8%, 4.6%)	0.837 0.805

Abbreviations: CI, confidence interval; ml, millilitre; POC, point‐of‐care; Q1, quartile one (25%); Q3, quartile three (75%); RD, risk difference; RR, relative risk; SOC, standard‐of‐care; VL, viral load.

^a^ Risk ratios, confidence intervals and *p*‐values generated by Poisson regression modified with robust standard error estimation, adjusting for facility only. *p*‐Values indicate statistical significance of the effect of the intervention on viral suppression of study participants.

^b^ Risk ratios, confidence intervals and *p*‐values generated by Poisson regression modified with robust standard error estimation, adjusting for facility, baseline ART regimen and baseline food insecurity. *p*‐Values indicate statistical significance of the effect of the intervention on viral suppression of study participants.

^c^ Risk ratios, confidence intervals and *p*‐values generated by Poisson regression modified with robust standard error estimation, adjusting for inverse probability weighting for missing outcomes. *p*‐Values indicate statistical significance of the effect of the intervention on viral suppression of study participants.

^d^ For intervention group participants, POC VL testing was intended to be performed at each study visit, which were targeted for every 12 weeks. For both intervention and control group participants, SOC VL testing was performed at each facility, which generally would be expected to be every 6 months. We used chart review as well as review of the Kenya Ministry of Health's NASCOP HIV VL Database to find available regular clinic VL testing both prior to enrolment and during the study. We assigned such available SOC VL test results and from POC VL testing to the visit window into which the blood draw fell (the target date +/− 6 weeks) except for baseline (0 months) which included up to 90 days prior to enrolment. Thus, we assigned the VL results as 0 months if blood was drawn 0−90 days prior to enrolment, 3 months if 1 day to 18 weeks after enrolment, 6 months if 18 weeks + 1 day to 30 weeks after enrolment, delivery if 2 weeks before to 6 weeks after the date of delivery, 3 months postpartum if 6 weeks + 1 day to 18 weeks after date of delivery and 6 months postpartum if 18 weeks + 1 day to 30 weeks after date of delivery. If more than one POC or SOC VL test result was available in any interval, the one closest to the target study visit date was used. If a POC VL test was not available at 6 months postpartum, any available SOC test in the same window was used; this occurred in 7/328 (2.1%) and 120/347 (34.6%) of instances for the primary outcome of 6 months postpartum VL assessment in the intervention and control groups, respectively.

^e^ Primary outcome of viral suppression of participants with samples collected at 6 months +/− 16 weeks (56−280 days) after delivery were assigned as the 6‐month postpartum VL. Our protocol defined endpoint was POC testing at 6 months postpartum for both intervention and control groups; however, if POC testing was not available in the interval 224−448 days from date of delivery, the SOC VL test result was used if available.

### 3.2 Primary outcome of VS proportions

At 6 months postpartum, 321/328 (98%) of participants in the intervention group and 339/347 (98%) in the control group achieved VS by POC VL testing (aRR 1.00, 95% confidence interval [CI] 0.98, 1.02; Table 2). When using a lower threshold of <400, results were almost identical; using <40 copies/ml, the VS proportion at 6 months postpartum was lower than seen with the higher thresholds but still similar by group. Findings from the sensitivity analyses, adjusting for differing enrolment characteristics between the groups and inverse probability weighting analysis, were similar (Table 2).

### 3.3 DRM testing, resistance identified and ART change recommendations

In the intervention group, from enrolment up until 6 months postpartum, we identified 54 episodes of VL>1000 copies/ml, among 48 (11.7%) participants (Table 3). In the intervention group, 52 DRM tests were requested, of which 46 (88%) were successfully conducted (six samples failed to amplify) and all identified at least one DRM (K103N [*n* = 12, 28%] and M184V [*n* = 10, 22%] were most commonly detected mutations). Our Clinical Management Committee recommended that 6 (12%) of the 48 women undergo an ART change, of which all six (100%) had an ART change documented by 6 months postpartum. In contrast, we recorded 37 episodes of VL>1000 copies/ml, among 29 (7%) control participants with no DRM tests requested in this group.

**Table 3:** Description of drug resistance testing up until 6 months postpartum of study follow‐up and outcomes by study group among Opt4Mamas study participants (*n*=820), February 2019−December 2021^a^

Variable	Intervention group (POC VL; *n*=411)	Control group (SOC VL; *n*=409)
*Episodes of viraemia (can be more than one per participant)*		
Episodes of viraemia (> 1000 copies/ml)^a^	54^b^	37^c^
Number of DRM test requested	52	0
Number of DRM test performed successfully	46/52 (88%)^d^	–
Turnaround time from time DRM tests requested to results returned to study staff (in days), median (IQR)	22 (16, 30)	–
Any DRM identified	46/46 (100%)	–
Any major DRMs identified^e^	20/46 (43%)	–
Any resistance type by HIV drug classes^f^		
NRTI	46/46 (100%)	–
NNRTI	46/46 (100%)	–
PI	46/46 (100%)	–
Major resistance type by HIV drug classes		
NRTI	11/20 (55%)	–
NNRTI	16/20 (80%)	–
PI	2/20 (10%)	–
ART change recommended per each DRM test successfully conducted	6/52 (12%)	–
Recommended ART change made by 6 months postpartum	6/6 (100%)	–

Abbreviations: ART, antiretroviral therapy; DRM, drug resistance mutation; IQR, interquartile range; NNRTI, non‐nucleos(t)ide reverse transcriptase inhibitor; NRTI, nucleos(t)ide reverse transcriptase inhibitor; PI, protease inhibitor; POC, point‐of‐care; SOC, standard‐of‐care; VL, viral load.

^a^ From date of study enrolment through any time point prior to the 6 months postpartum study visit (e.g. data exclude results obtained as part of postpartum 6 study visit).

^b^ Forty‐eight participants had a total of 54 viraemic episodes detected (5 of these 48 [10%] participants had repeat viraemic episodes).  Of these 54 samples, we did not request DRM testing for two because of insufficient sample, resulting in our requesting DRM test for 52 samples.

^c^ Twenty‐nine participants had a total of 37 viraemic episodes detected.

^d^ Out of 52 samples where we requested DRM test, six (12%) samples failed to amplify.

^e^ We define major classification for HIV drug resistance according to the Stanford HIV Resistance Database.

^f^ The most commonly detected DRM by HIV drug class included: (1) NRTI—M184V (*n*=10 DRM tests; detected in 22% of all DRM tests resulted), K70R/Q (7; 15%), and one (2%) each of K65R, D67N, L74I, V75M and K219R; (2) NNRTI—K103N (*n*=12; 28%), V108I (3; 7%), P225H (3; 7%), G190A/S (2; 4%), E138A/G (2; 4%), K238T (2; 4%), and one (2%) each of K101E, V106I and Y181C; and (3) PI—L89M (32; 70%), I13V (28; 61%), and one (2%) each of L24I, L33F, K43T, M46L, I54V and V82A.

### 3.4 Secondary outcomes of VL testing uptake, turnaround time and infant testing

Of the participants attending each study visit in the intervention group, 100%, 51%, 43%, 90%, 75% and 94% had a POC VL conducted at 0, 3 and 6 months after enrolment, delivery, 3 and 6 months postpartum, respectively (Table 4); the 3‐ and 6‐month after enrolment visits were heavily impacted by COVID‐19‐related restrictions in 2020. Among participants in the control group, 66%, 27%, 15%, 0%, 38% and 42% had a SOC VL conducted at 0, 3 or 6 months after enrolment, delivery, and at 3 and 6 months postpartum, respectively.

**Table 4:** Process measures regarding point‐of‐care viral load and drug resistance testing among Opt4Mamas study participants (*n*=820), February 2019−December 2021

	Intervention group (POC VL; *n*=411)	Control group (SOC VL; *n*=409)
Participants attending the study visit among those expected^a^		
0 months	411/411 (100%)	409/409 (100%)
3 months	283/296 (96%)	288/301 (96%)
6 months	53/70 (76%)	78/85 (92%)
Delivery	345/380 (91%)	367/376 (98%)
Postpartum 3	276/373 (74%)	342/368 (93%)
Postpartum 6	321/364 (88%)	333/361 (92%)
*Subtotal from 0 to postpartum 6*	1689/1894 (89%)	1817/1900 (96%)
Postpartum 9+	34/2148 (2%)	288/2071 (14%)
*Total from 0 to postpartum 24*	1723/4042 (43%)	2105/3971 (53%)
POC VL test conducted^b^ for intended study visit among participants attending the study visit		
0 months	409/411 (99.5%)	
3 months	143/283 (50.5%)	
6 months	23/53 (43.4%)	
Delivery	309/345 (89.6%)	16/367 (4.6%)
Postpartum 3	206/276 (74.6%)	
Postpartum 6	301/321 (93.8%)	255/333 (76.6%)
*Subtotal from 0 to postpartum 6*	1391/1689 (82.4%)	271/700 (38.7%)
Postpartum 9+	28/34 (82.4%)	
*Total from 0 to postpartum 24*	1419/1723 (82.4%)	271/700 (38.7%)
SOC VL test conducted^b^ within testing interval among participants regardless of attending the study visit		
0 months	156/411 (38%)	268/409 (65.5%)
3 months	40/411 (9.7%)	111/409 (27.1%)
6 months	10/411 (2.4%)	60/409 (14.7%)
Delivery		
Postpartum 3	26/411 (6.3%)	146/409 (35.7%)
Postpartum 6	17/411 (4.1%)	173/409 (42.3%)
*Subtotal from 0 to postpartum 6*	249/2055 (12.11%)	758/2045 (37.1%)
*Postpartum 9 + months*	16/1233 (1.3%)	289/1636 (17.7%)
*Total from 0 to 24 months*	265/3288 (8.1%)	1047/3681 (28.4%)
Either POC for intervention group or SOC for control group VL test returned to participant/caregiver, and within 24 hours of blood draw^b^		
0 months	391/409 (95.6%), 334/409 (81.7%)	Data not available
3 months	131/143 (91.6%), 106/143 (74.1%)	Data not available
6 months	19/23 (82.6%), 16/23 (69.6%)	Data not available
Delivery	281/309 (91.0%), 245/309 (79.3%)	Data not available
Postpartum 3	192/206 (93.2%), 156/206 (75.7%)	Data not available
Postpartum 6	291/301(96.7%), 93/301 (30.9%)	Data not available
*Subtotal from 0 to postpartum 6*	1305/1391 (93.8%), 494/816 (60.5%)	Data not available
*Postpartum 9 + months*	28/28 (100%), 15/26 (57.7%)	Data not available
*Total from 0 to 24 months*	1333/1419 (94%), 509/842 (60.5%)	Data not available
POC VL test returned to participant, and within 24 hours of blood draw		
0 months	391/409 (95.6%), 334/409 (81.7%)	
3 months	131/143 (91.6%), 106/143 (74.1%)	
6 months	19/23 (82.6%), 16/23 (69.6%)	
Delivery	281/309 (91.0%), 245/309 (79.3%)	10/16 (62.5%), 9/16 (56.3%)
Postpartum 3	192/206 (93.2%), 156/206 (75.7%)	
Postpartum 6	291/301 (96.7%), 93/301 (30.9%)	237/255 (93.0%), 189/255 (74.1%)
*Subtotal from 0 to postpartum 6*	1305/1391 (93.8%), 494/816 (60.5%)	247/271 (91.1%), 198/205 (96.6%)
Postpartum 9+	28/28 (100%), 15/26 (57.7%)	
*Total from 0 to 24 months*	1333/1419 (94%), 509/842 (60.5%)	247/271 (91.1%), 198/205 (96.6%)
Either POC or SOC VL test returned to provider, and within 24 hours of blood draw^c^		
0 months	391/409 (95.6%), 331/391 (84.7%)	268/268 (100%)
3 months	132/143 (92.3%), 107/132 (81.1%)	111/111 (100%)
6 months	19/23 (82.6%), 16/19 (84.2%)	60/60 (100%)
Delivery	281/309 (90.1%), 242/281 (86.1%)	
Postpartum 3	192/206 (93.2%), 153/192 (79.7%)	146/146 (100%)
Postpartum 6	291/301 (96.7%), 93/291 (32.0%)^d^	173/173 (100%)
*Subtotal from 0 to postpartum 6*	1306/1391 (93.9%), 942/1306 (72.1%)	758/758 (100%)
*Postpartum 9 + months*	28/ 28 (100%), 15/28 (53.6%)	289/289 (100%)
*Total from 0 to 24 months*	1334/1419 (94%), 957/1334 (71.7%)	1047/1047 (100%)
Number of POC VL test returned to provider, and within 24 hours of blood draw		
0 months	391/409 (95.6%), 331/391 (84.7%)	
3 months	132/143 (92.3%), 107/132 (81.1%)	
6 months	19/23 (82.6%), 16/19 (84.2%)	
Delivery	281/309 (90.1%), 242/281 (86.1%)	10/16 (62.5%), 9/10 (90.0%)
Postpartum 3	192/206 (93.2%), 153/192 (79.7%)	
Postpartum 6	291/301 (96.7%), 93/291 (32.0%)^d^	237/255 (93.0%), 184/237 (77.6%)
*Subtotal from 0 to postpartum 6*	1306/1391 (93.9%), 942/1306 (72.1%)	247/271 (91.1%), 193/247 (78.1%)
*Postpartum 9 + months*	28/ 28 (100%), 15/28 (53.6%)	
*Total from 0 to 24 months*	1334/1419 (94%), 957/1334 (71.7%)	247/271 (91.1%), 193/247 (78.1%)
Median (IQR) turnaround time in days for VL requested (from sample collection to result return to provider), by POC VL testing for intervention group and SOC VL testing for control group	1 (1, 4)	7 (5, 9)
Number of VL tests from enrolment to 6 months postpartum (POC VL for intervention group, SOC VL for control group)		
At least one	406 (98.8%)	393 (96.1%)
At least two	358 (87.1%)	351 (85.8%)
Median (IQR)	4 (2, 5)	3 (2, 3)
Number of VL tests from enrolment to delivery (POC VL for intervention group, SOC VL for control group)		
At least one	401 (97.6%)	369 (90.2%)
At least two	305 (74.3%)	187 (45.7%)
Median (IQR)	2 (2, 3)	2 (1, 2)
Number of VL tests from delivery to 6 months postpartum (POC VL for intervention group, SOC VL for control group)		
At least one	343 (83.5%)	355 (86.8%)
At least two	238 (58.0%)	183 (44.7%)
Median (IQR)	2 (1, 3)	2 (1, 2)

Abbreviations: IQR, interquartile range; PI, protease inhibitor; POC, point‐of‐care; SOC, standard‐of‐care; VL, viral load.

^a^ We define participants attending the study visit as those completing study questionnaires, though not necessarily in‐person, among those expected to attend the study visit (i.e. retention in study).

^b^ VL tests were considered to have been conducted if a sample was collected for testing and sent for VL testing.

^c^ Because VL test results are only tracked by results released to the local laboratory in the Kenya Ministry of Health's NASCOP HIV VL database, we are not able to track how many VL tests were requested versus those finally resulted. Thus, the results returned are 100% for SOC.

^d^ Of note, our study encountered a 3‐month delay in being able to test our study samples via POC VL testing due to the global reagent shortages experienced during the COVID‐19 pandemic.

Of the POC VL tests conducted in the intervention group during the entire study period, 90% were returned to the participant, and ≥ 60% were returned within 24 hours of the blood draw (excluding the 6‐month postpartum visit in which only 31% were returned within 24 hours due to disruptions in the global supply of POC VL cartridges for GeneXpert systems; Table 4). Return of results to providers followed similar patterns. Neither the number of total VL test requests nor the turnaround time from sample collection to return of results to the women was available in the control group. From sample collection to result return to providers, the median turnaround time was 1 day (IQR 1, 4) for POC VL testing in the intervention group and 7 days (IQR 5, 9) for SOC VL testing in the control group.

Through the 6‐month postpartum visit, one and three infants tested HIV positive in the intervention and control groups, respectively (Table S5).

### 3.5 Secondary outcome of sustained VS

SVS was lower than VS point prevalence at 6 months postpartum but was still similar by group; 311/352 (88.4%) in intervention group and 332/366 (90.7%) in control group (aRR 0.98, 95% CI 0.93, 1.03) (Table 5). SVS measured at <400 copies/ml was 304/352 (86.4%) and 321/366 (87.7%; aRR 0.97, 95% CI 0.91, 1.02) and at <40 copies/ml was 246/352 (70.0%) and 226/366 (61.7%; aRR 1.00, 95% CI 0.89, 1.12) in the intervention and control groups, respectively.

**Table 5:** Effect of the intervention on sustained^a^ viral suppression for all study intervals through 6‐month postpartum visit for every participant with two or more VL test results in two separate study intervals, and varying threshold of VL cutoffs among the Opt4Mamas study participants (*n*=820), February 2019−December 2021

Variable	Intervention (*n*=411)	Control (*n*=409)	Unadjusted RR^b^ (95% CI)	*p*‐Value	Adjusted RR^b^ (95% CI)	*p*‐Value
Viral suppression < 1000 copies/ml	311/352 (88.4%)	332/366 (90.7%)	0.97 (0.92, 1.02)	0.213	0.98 (0.93, 1.03)	0.378
Viral suppression < 400 copies/ml	304/352 (86.4%)	321/366 (87.7%)	0.97 (0.91, 1.02)	0.256	0.97 (0.91, 1.02)	0.239
Viral suppression < 40 copies/ml	246/352 (70.0%)	226/366 (61.7%)	1.03 (0.92, 1.15)	0.587	1.00 (0.89, 1.12)	0.958

Abbreviations: POC, point‐of‐care; SOC, standard‐of‐care; VL, viral load.

^a^ Sustained viral suppression is defined as having viral load less than viral load cutoff in all viral load tests. For instance, for viral load cutoff <1000 copies/ml, a participant is sustained virally suppressed if the participant has viral load <1000 copies/ml in all viral load tests taken.

^b^ Risk ratio of intervention adjusted for number of tests.

## 4 DISCUSSION

In this prospective, intervention/control cohort study among women living with HIV on ART, we did not observe differences in VS between women receiving a combined intervention with POC with higher frequency VL testing every 3 months, targeted DRM testing, and clinical decision support, and control women, during pregnancy, delivery or the postpartum periods. Overall, we observed >90% VS at 6 months postpartum in this study at thresholds of VL <1000, <400 and <40 copies/ml. However, a sizeable proportion of women experienced viraemia during pregnancy and postpartum periods when considering all VL checks (10−38%), and major DRMs among these women may be important. POC VL and DRM testing was highly feasible in this setting, with rapid turnaround times, and resulted in a larger proportion of women undergoing these testing during pregnancy and postpartum periods.

We did not observe any associations between our combined intervention and VS among pregnant and postpartum women. Our failure to demonstrate efficacy may be due to biased sampling of women better engaged in care, inability to implement POC VL testing with optimal fidelity, overall improvements in VS among the women over time and/or the increased use of dolutegravir‐containing ART which is equally or more potent than efavirenz‐containing ART [37−39]. Nonetheless, when considering SVS throughout the pregnancy and postpartum periods, a sizeable number of women lacked VS at some point during pregnancy, as low as 62% when using the lowest VL threshold of <40 copies/ml. With maternal VL being the greatest predictor of vertical transmission, achieving VS among pregnant and postpartum women remains an enduring concern; vertical transmission rates were around 2% among mothers with VLs ranging from 40 to 1000 copies/ml [3]. In our study, few vertical transmissions were recorded and POC VL uptake was high. Efforts to eliminate vertical transmission will require rapid identification and intervention for pregnant and breastfeeding women with viraemia, and POC VL may still have a targeted role in achieving the elimination of vertical transmission.

The evidence base regarding the utility of POC VL testing in improving clinical outcomes is mixed. We saw no efficacy on VS in our parallel, randomized controlled trial in children [22]. A South African randomized controlled trial compared 3‐monthly POC VL testing to 6‐monthly SOC laboratory‐based VL testing among postpartum women living with HIV on first‐line ART, and found no significant difference in VL suppression rates [23]. Similarly, a study among Nigerian adults initiating ART reported that POC VL monitoring did not improve 12‐month VS, but it did improve retention and VS documentation and was favoured by the majority of patients and healthcare workers [24]. Other studies demonstrated some benefits to POC VL. A preliminary analysis suggests significant improvement in VS (7%) among pregnant/breastfeeding Ugandan women, children/adolescents (2–17 years), viraemic patients and patients overdue for VL who received POC VL [25]. A study that combined POC VL with a differentiated service delivery strategy resulted in enhanced VS by 10.3% and retention by 7.7% among South African adults living with HIV [26]. Ultimately, despite some studies showing no efficacy, enthusiasm for POC VL testing exists not only among patients and providers, but also among policymakers at the national and international levels [18, 32, 40]. Future research needs to help elucidate cost‐effective ways for when best to use POC VL testing, among whom and at what interval frequency.

Among women with viraemia in our study who underwent successful DRM testing, all had some DRMs and 43% had major DRMs (with NNRTI K103N and NRTI M184V being the most common). A study conducted in Sierra Leone showed K103N as the most frequent DRM, occurring in 20% of pregnant women, with M184V in 11% [41]. Among Kenyan pregnant women, 65% of viraemic women showed some DRMs [42]. Among women initiating ART, most of whom reported prior exposure to antiretrovirals for prevention of vertical transmission, the prevalence of DRM to NNRTIs was 14.6% [28]. INSTI resistance testing was unavailable in Kenya during the study period, and while greater than half of the women transitioned to INSTI‐containing regimens by the study end, we do not think the inclusion of INSTI resistance testing would have altered our overall findings, as the emergence of dolutegravir resistance with short exposures is unlikely [43]. However, the inclusion of INSTI resistance testing will become necessary as women become viraemic after a longer duration on dolutegravir‐containing regimens. In the current study, around one in eight women with a DRM test result required ART change; at a population level, this is a substantial number. Lack of drug resistance may be equally valuable for clinicians as this indicates that the regimen does not require a switch for resistance reasons and suggests other causes for viraemia. From our clinical case reviews, it appears that many women with viraemia face larger, psychosocial and behavioural challenges (manuscript forthcoming) which require additional interventions in combination with information provided by DRM testing. Thus, differentiated service delivery models need to be urgently developed to concentrate the needed extra resources for those women struggling with VS, including women on salvage regimens or not engage in care (noting such women were excluded from our study), during this dynamic period of their pregnancy care.

### 4.1 Limitations

Ours is the first study to combine VL with DRM testing in optimizing VS among pregnant and postpartum women; however, it has limitations. First, Opt4Mamas could not be pursued as a randomized clinical trial as a limitation of its funding source, but we believed a contemporaneous intervention/control study, which had overlapping cohorts in time, was the next best robust study design to pursue. Second, our combined strategy of multiple interventions precludes the determination of the success or failure of any one component of the strategy. Additionally, layering on POC VL testing on top of routine care, where providers were not prevented from ordering routine SOC VL testing, further limits the interpretation of our findings. Third, some spillover effects from intervention to control participants may have occurred. Though our intervention package was only offered to intervention group participants, it was the same providers caring for control group participants. We observed greater fidelity in conducting SOC VL tests in the control group than expected and, anecdotally, increased confidence in facility staff over time in managing women with viraemia. Our randomized trial in children faced this same issue [22]. Alternative study designs, such as facility‐level cluster randomization, may avoid potential spillover effects though would require greater resources. Fourth, we note that VS was assessed more frequently in the intervention versus control group, so, theoretically, the intervention group participants had a greater number of opportunities to act on their test results but also greater opportunities to detect viraemia. Ultimately, restrictions and reagent stockouts related to COVID‐19 greatly compromised our ability to conduct POC VL testing every 3 months or return results within 24 hours as planned. Thus, it is possible that intervention group participants did not receive sufficient POC VL testing to impact their outcomes. While the impacts of COVID‐19 may not be as significant in the future, it is likely that similar programmatic challenges will persist. Lastly, potential measurement bias in intention‐to‐treat estimates and selection bias due to missing outcome data (on approximately 15–20% of our enrolled participants) are limitations; however, our sensitivity analysis using inverse probability weighting substantiated our primary outcome analysis findings.

## 5 CONCLUSIONS

In a prospective cohort study with an intervention/control study design with the intervention consisting of a combined strategy of POC with higher frequency VL testing, targeted DRM testing and clinical decision‐making support versus SOC, we observed high rates of VS at 6 months postpartum in both groups and no difference between the intervention and control groups during pregnancy, at delivery or postpartum. Nonetheless, a sizeable number of women experienced viraemia, when considering SVS throughout pregnancy and postpartum periods, many of whom had major DRMs. POC VL uptake was high and DRM testing was feasible. Ultimately, it remains unclear what interventions pregnant/postpartum women with viraemia need to optimize VS, their health outcomes and help prevent vertical transmission.

## COMPETING INTERESTS

The authors declare no competing interests.

## AUTHORS’ CONTRIBUTIONS

Conceptualization—RCP, LLA, PO, IM and KKT.

Data curation—NY and KKT.

Access and verified data—NY, GWB and KKT.

Formal analysis—GWB, NY, KKT, RCP, LLA and PO.

Funding acquisition—RCP.

Investigation—RCP, LLA, PO, IM, KKT, JW, LK and EK.

Methodology—RCP, LLA, PO and KKT.

Project administration—EB, PO, KKT, SAH, BO, LK, EK, NY, RCP and LLA.

Resources—PO, KKT, JW, EK, FO, FO, BO, LK, EK, NY, LO, RCP and LLA.

Software—GWB, NY, KKT, BO and LK.

Supervision—EB, PO, KKT, JW, EK, FO, FO, BO, LK, EK, NY, LO, RCP, LLA and GCJ‐S.

Validation—N/A.

Visualization—GWB, NY, KKT and SAH.

Writing, original draft—RCP, LLA, PO, KKT, GWB and SAH.

Writing, review and editing—all coauthors.

Decision to submit manuscript—RCP, LLA, PO and KKT.

## FUNDING

This work was supported by the National Institutes of Allergy and Infectious Diseases of the U.S. National Institutes of Health (NIH, CFAR NIA 2P30AI027757‐31 and R21 R21AI145450). Study data were collected and managed using REDCap electronic data capture tools hosted at the University of Washington Institute of Translational Health Sciences and supported by the National Center for Advancing Translational Sciences of the NIH (UL1 TR002319).

## DISCLAIMER

The funding sources or study sponsors had no role in study design; in the collection, analysis and interpretation of data; in the writing of the report; and in the decision to submit the paper for publication.

### ETHICAL REVIEW STATEMENT

Ethical approval for this study has been obtained from the African Medical and Research Foundation (AMREF) and Jaramogi Oginga Odinga Teaching and Referral Hospital (JOOTRH) Institutional Review Boards (IRBs) in Kenya, as well as the University of Washington and the University of Colorado Denver IRBs in the United States.  All study procedures were performed in accordance with the Declaration of Helsinki.

## Supporting information

Supplementary Text

Supplemental Figure 1

Supplemental Tables

## Data Availability

De‐identified participant data, data dictionary or other specified data sets may be made available to others requesting them upon communication with corresponding author, demonstration of appropriate ethic reviews and establishment of data sharing agreements. Study protocol, statistical analysis plan, informed consent forms, analysis code or other documents can be made available upon request with the corresponding author.
