# Supplementary material for: Impact of point‐of‐care HIV viral load and targeted drug resistance mutation testing on viral suppression among Kenyan pregnant and postpartum women: results from a prospective cohort study (Opt4Mamas)
Source: J Int AIDS Soc. 2023 Nov 8;26(11):e26182. doi: 10.1002/jia2.26182 (PMC10631517; doi:10.1002/jia2.26182)
Supplement: Supplementary file 1 — Supplementary Text [file JIA2-26-e26182-s003.docx]

**Supplementary Text**

**2.0 Methods (cont.)**

**2.6 Study procedures**

*Enrollment, allocation, and blinding*

Study staff at each facility approached potential participants for study participation at routine clinical visits and obtained written informed consent. Staff enrolled participants in the control group until 410 participants were enrolled, after which participants were enrolled to the intervention group, until another 410 participants were enrolled. Of note, follow-up of the control cohort overlapped in calendar time with enrollment and follow-up of the intervention cohort. Investigators were blinded to the group allocation, while participants, research field staff, and the data team were not. A total of eight protocol deviations occurred, including four instances of control group participants’ samples undergoing intervention testing procedures, two instances of participants being enrolled twice, and one instance each of a participant being enrolled after 32 weeks of gestation and another one self-reporting not pregnant on repeat pregnancy testing (**Figure 1**).

*2.6.1 Control- SOC VL and DRM testing*

Kenya Ministry of Health (MOH) guidelines during the study period recommended VL monitoring at 1^st^ ANC visit if already on ART or at 6 months post-ART initiation for newly diagnosed women, followed by VL testing every 6 months postpartum while breastfeeding.^1,2^ SOC VL testing was conducted by two routine care laboratories for the participating facilities. Quality assurance testing at these facilities followed routine care laboratory procedures, which included quarterly internal specimen testing. Generally, once the sample is processed, the results from the centralized SOC VL laboratories are uploaded into the national database maintained by the Kenya NASCOP.^3^ The hub laboratory personnel periodically log into the website to check/track and print results and then pass them to the spoke facilities via designated riders/drivers. At the treatment facilities, the results are recorded at the facility VL register/tracking log and placed in the patient file or the information is updated into the electronic medical record. Clinical providers can also routinely access available results via short text messaging system or an online platform and not use the hard copy results. Results that show lack of suppression are flagged at the facility prompting tracking of the patients for a quicker return before the documented return date. Management of pregnant/postpartum women with VL >1000 copies/mL included enhanced adherence counseling and repeat VL testing after three months of good adherence followed by switch of ART to second-line regimens if still not virologically suppressed. DRM testing at national reference laboratories became accessible in 2018 but was restricted to adults with virologic failure on 2^nd^ or 3^rd^ line ART who continued to have viremia after adherence optimization. National SOC DRM testing required approvals by regional Kenyan MOH HIV technical working groups who guided local providers on clinical management.

*2.6.2 Intervention- POC VL testing*

Our POC VL testing approach utilized the existing GeneXpert^®^ system (which can simultaneously perform TB and HIV diagnostics) at four facilities. For the facility without a GeneXpert^®^ system on site, samples were transported daily to a facility less than 2km away. POC VL testing was conducted by routine laboratory staff. The study participated in a quarterly external quality assurance program for the POC VL testing platform, where external controls with known quantification levels were tested and each facility passed each check. Fidelity to the group allocations was maintained by restricting POC VL test ordering to study staff.

We conducted POC VL testing at study enrollment and then every three months while the women remained pregnant, at delivery, and then every three months postpartum. Results were delivered via text message or phone to participants and paper results for providers. This VL testing schedule was more frequent than the SOC schedule in Kenya and was intended to inform more rapid clinical management and participant adherence behavior through VL result counseling leading to improved VS. Of note, SOC VL testing per routine care could be continued by clinical facility staff in intervention group participants; thus, in some ways, POC VL testing could be layered on top of routine care SOC VL testing.

*2.6.3 Intervention- targeted HIV DRM testing*

Targeted HIV DRM testing was performed on plasma samples with VL > 1000 copies/ml using Sanger sequencing with Applied Biosystems 3130xl Genetic Analyzers at the KEMRI-CDC HIV Research and Sanger 3730xl at the Kenya National HIV Reference Laboratories. These laboratories were WHO accredited for Sanger sequencing and conducted routine laboratory quality control/assurance procedures, including specimen rejection criteria (e.g., hemolysis, clotting, or specimen identification mismatch), periodic phylogenetic mapping of sequences to ensure prevention of contamination or specimen mix-up, and control testing to ensure detection of contamination or other issues. Specific sequence quality assessments were conducted by study team for study specimens prior to data interpretation. Subsequent episodes of VL > 1000 copies/ml did not necessarily trigger a DRM test if on the same ART regimen (initial protocol did not plan to repeat DRM, but it was later changed to allow repeat DRMs (to allow detection of accumulation of additional mutations) even if on the same regimen, and given low samples sizes for repeat DRMs in this study, we did not pursue analyses based on repeat DRM). Integrase inhibitor testing was not conducted due to the testing laboratories not having WHO certification yet for the procedures needed to test for such resistance. The laboratories conducted batch testing at periodic intervals and returned results to the study staff within 24 hours of assay result. Study staff then forwarded the DRM results to clinical providers within 24 hours.

*2.6.4 Intervention- clinical decision support for management of women on ART with drug resistance*

For both groups, clinical providers were instructed to follow current Kenyan national guidelines for managing pregnant/postpartum women with VL >1000 copies/mL. For the intervention group, a Clinical Management Committee (CMC) was formed based on the existing MOH regional technical working group. The committee included the regional technical working group chair, facility clinical and psychosocial service providers, study staff, principal investigators, MOH and other country HIV experts, and HIV implementing partner technical advisors. It met at least monthly to conduct case reviews of every participant with a DRM result using a standardized case review form prepared by facility and study staff. ART regimen changes were highly individualized with available antiretrovirals in Kenya, including from existing 1^st^ or 2^nd^ line ART regimens and namely dolutegravir- or atazanavir/ritonavir-containing regimens. Recommendations regarding ART regimen and case management were agreed on by consensus and summarized using a standardized CMC recommendation form.

*2.6.5 Retention activities*

Clinic-based retention activities included text message reminders, phone calls, home visits, and loss-to-follow-up tracing by clinic staff, which research staff supplemented with additional phone calls or text messaging as needed. We provided 500 Kenyan Shillings (approximately USD 5) to the participant for enrollment and primary endpoint (6 months postpartum) study visits and a delivery visit gift package (consisting of diapers, soap, and baby clothes, costing approximately USD 10).

*2.6.6 Data collection*

Our data collection included in-person (or via phone during periods of the COVID-19 pandemic) questionnaires, review of routine MOH standardized patient records, including paper or electronic medical records, for clinical and laboratory information, and sample collection when applicable.

**2.7 Patient and public involvement**

Participants, patients at the same facilities, providers, and other stakeholders were invited to in-person or virtual findings dissemination meetings to provide formative feedback in improving our final analyses and interpretations.

**2.8 Ethical review statement**

Ethical approval for this study has been obtained from the African Medical and Research Foundation (AMREF) and Jaramogi Oginga Odinga Teaching and Referral Hospital (JOOTRH) Institutional Review Boards (IRBs) in Kenya, as well as the University of Washington and the University of Colorado Denver IRBs in the United States.  All study procedures were performed in accordance with the Declaration of Helsinki.

References

1. Kenya Ministry of Health M. Guidelines for prevention of Mother to Child Transmission (PMTCT) of HIV/AIDS in Kenya 4th Edition, 2012. 2012. (<http://guidelines.health.go.ke:8000/media/Guidelines_for_PMTCT_of_HIVAIDS_in_Kenya-1.pdf>).

2. Ministry of Health Kenya NASCOP framework for elimination of mother-to-child transmission of HIV and syphilis 2016-2021. . Ministry of Health (MOH); 2016. Nairobi, Kenya2016.

3. Programme NAaSC. Kenya NASCOP Viral load dashboard. (<https://viralload.nascop.org>).
