# Supplementary material for: Impact of point‐of‐care HIV viral load and targeted drug resistance mutation testing on viral suppression among Kenyan pregnant and postpartum women: results from a prospective cohort study (Opt4Mamas)
Source: J Int AIDS Soc. 2023 Nov 8;26(11):e26182. doi: 10.1002/jia2.26182 (PMC10631517; doi:10.1002/jia2.26182)
Supplement: Supplementary file 2 — Supplemental Figure 1 [file JIA2-26-e26182-s002.pptx]

## Slide 1
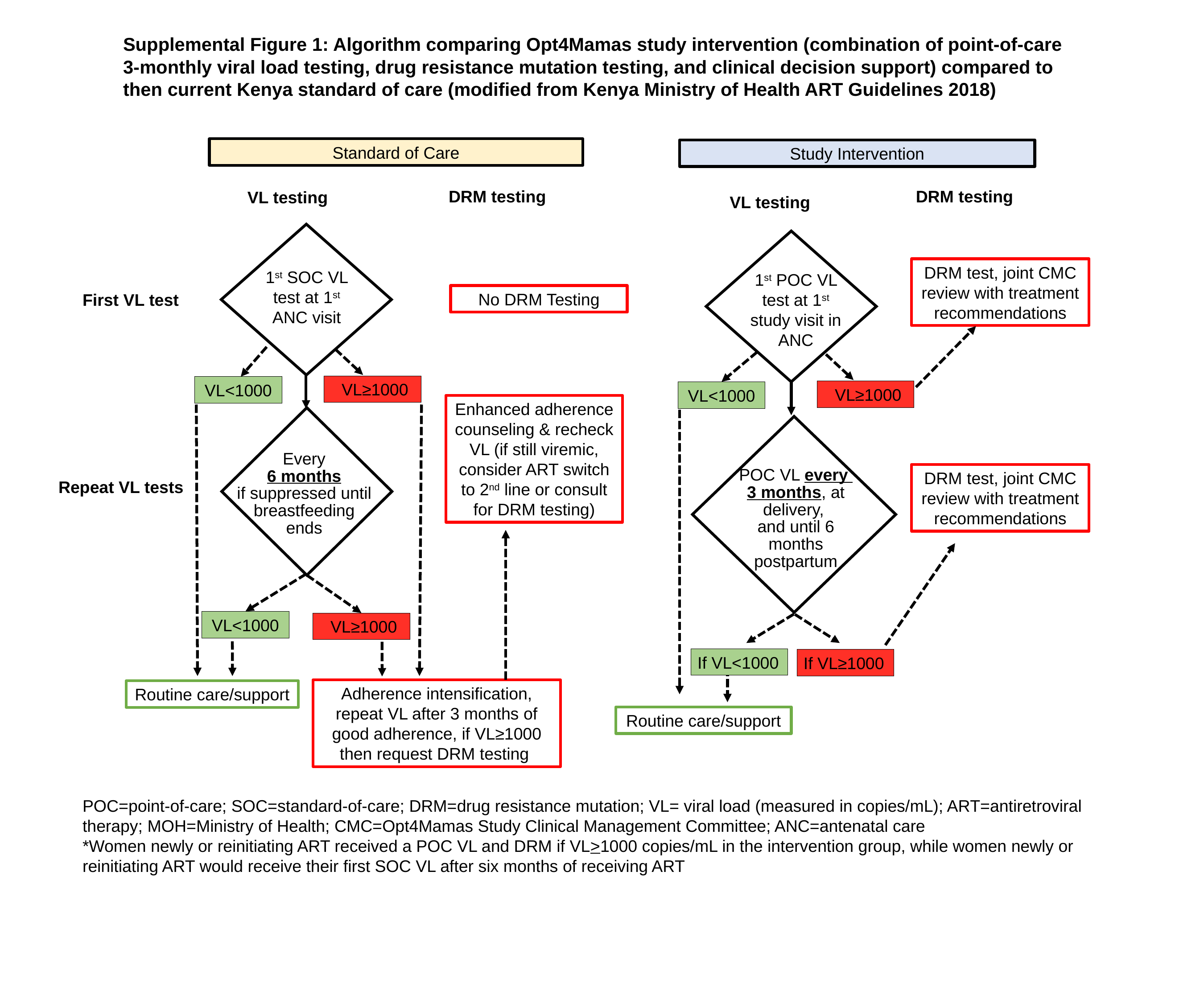

Supplemental Figure 1: Algorithm comparing Opt4Mamas study intervention (combination of point-of-care 3-monthly viral load testing, drug resistance mutation testing, and clinical decision support) compared to then current Kenya standard of care (modified from Kenya Ministry of Health ART Guidelines 2018)
Standard of Care
Study Intervention
DRM testing
DRM testing
VL testing
VL testing
DRM test, joint CMC review with treatment recommendations
1st SOC VL test at 1st ANC visit
1st POC VL test at 1st study visit in ANC
No DRM Testing
First VL test
 VL≥1000
VL<1000
 VL≥1000
VL<1000
Enhanced adherence counseling & recheck VL (if still viremic, consider ART switch to 2nd line or consult for DRM testing)
Every
 6 months
if suppressed until breastfeeding ends
POC VL every
3 months, at delivery,
and until 6 months postpartum
DRM test, joint CMC review with treatment recommendations
Repeat VL tests
VL<1000
 VL≥1000
If VL<1000
If VL≥1000
Adherence intensification, repeat VL after 3 months of good adherence, if VL≥1000 then request DRM testing
Routine care/support
Routine care/support
POC=point-of-care; SOC=standard-of-care; DRM=drug resistance mutation; VL= viral load (measured in copies/mL); ART=antiretroviral therapy; MOH=Ministry of Health; CMC=Opt4Mamas Study Clinical Management Committee; ANC=antenatal care
*Women newly or reinitiating ART received a POC VL and DRM if VL>1000 copies/mL in the intervention group, while women newly or reinitiating ART would receive their first SOC VL after six months of receiving ART
