# Supplementary material for: Impact of point‐of‐care HIV viral load and targeted drug resistance mutation testing on viral suppression among Kenyan pregnant and postpartum women: results from a prospective cohort study (Opt4Mamas)
Source: J Int AIDS Soc. 2023 Nov 8;26(11):e26182. doi: 10.1002/jia2.26182 (PMC10631517; doi:10.1002/jia2.26182)
Supplement: Supplementary file 3 — Supplemental Tables [file JIA2-26-e26182-s001.docx]

**SUPPLEMENTAL TABLES**

**Supplemental Table 1. Facility characteristics for five participating sites in study**

| **Variable** | **N (%) for facility (n=5)** | **N (%) for study participants (n= 820)** |
| --- | --- | --- |
| Facility type distribution  Teaching and referral hospital  County hospital  Sub-County hospital | 1 (20%)  3 (60%)  1 (20%) | 128 (16%)  612 (75%)  80 (10%) |
| Facility location type  Rural  Semi-urban  Urban | 1 (20%)  1 (20%)  3 (60%) | 80 (10%)  168 (20%)  572 (70%) |
| Facility average numbers of all patients seen for any outpatient visit, per month for 2019  Lumumba Subcounty Hospital  Kisumu County Hospital  JOOTRH  Ahero Subcountry Hospital  Nyakach Subcountry Hospital | 12894 (18%)  18919 (26%)  27155 (38%)  9051 (12%)  4298 (6%) | N/A |
| Facility average number of all PLWH seen for an outpatient visit, per month for 2019  Lumumba Subcounty Hospital  Kisumu County Hospital  JOOTRH  Ahero Subcountry Hospital  Nyakach Subcountry Hospital | 7209 (28%)  6302 (25%)  6558 (26%)  3589 (14%)  1964 (8%) | 149 (18%)  295(36%)  128(16%)  168(20%)  80(10%) |
| Facility average number of CLWH (age 1-14 years) seen for an outpatient visit, per month for 2019  Lumumba Subcounty Hospital  Kisumu County Hospital  JOOTRH  Ahero Subcountry Hospital  Nyakach Subcountry Hospital | 391 (26%)  391 (26%)  360 (24%)  237 (16%)  147 (10%) | N/A |
| On-site GeneXpert  Yes  No | 4 (80%)  1 (20%) | 671 (82%)  149 (18%) |

Abbreviations: PLWH=people living with HIV; JOOTRH=Jaramogi Oginga Odinga Teaching and Referral Hospital

**Supplemental Table 2: ART distribution over study visit time in the Opt4Mamas study, February 2019- August 2021**

|  | **At enrollment**  **(n=820)** | **Enrollment + 3 months**  **( n=597)** | **Enrollment + 6 months**  **(n=156)** | **Delivery**  **(n=756)** | **Postpartum + 3 months**  **(n=742)** | **Postpartum + 6 months**  **(n=717)** |
| --- | --- | --- | --- | --- | --- | --- |
| ART regimen distribution for all study participants, regardless of group n (%)  NNRTI-containing  PI-containing  Integrase-containing  Missing | 622 (75.9%)  71 (8.7%)  80 (9.8%)  47 (5.7%) | 397 (66.5%)  56 (9.4%)  112 (18.8%)  32 (5.4%) | 98 (62.8%)  15 (9.6%)  39 (25.0%)  4 (2.6%) | 406 (53.7%)  68 (9.0%)  236 (31.2%)  46 (6.1%) | 268 (36.1%)  70 (9.4%)  360 (48.5%)  44 (5.9%) | 152 (21.2%)  65 (9.1%)  459 (64.0%)  41 (5.7%) |

**Supplemental Table 3: VS^1^ (by either POC or SOC VL testing) and ART distribution over study visit time in the Opt4Mamas study, February 2019- August 2021**

|  | **At enrollment, VS (%)** | **Enrollment + 3 months** | **Enrollment + 6 months** | **Delivery** | **Postpartum + 3 months** | **Postpartum + 6 months** |
| --- | --- | --- | --- | --- | --- | --- |
| ART regimen distribution for all study participants, regardless of group n (%)  NNRTI-containing  PI-containing  Integrase-containing | 567/606(93.6%)  60/70(85.7%)  69/78(88.5%) | 331/341(97.1%)  51/54(94.4%)  96/103(93.2%) | 69/72(95.8%)  8/10(80.0%)  27/27(100%) | 321/330(97.3%)  53/58(91.4%)  182/185(98.4%) | 133/138(96.4%)  34/40(85.0%)  179/182(98.4%) | 24/25(96.0%)  13/13(100%)  84/86(97.7%) |
| Abbreviations:  ^1^Viral suppression (VS) is having viral load (VL) defined as <1000 copies/milliliter of blood. Participants having either POC and/or SOC VL test result in the study visit have been included in the analysis, and if both were available we used the POC VL test result. | | | | | | |

**Supplemental Table 4: Pregnancy outcomes in the Opt4Mamas study, February 2019- August 2021**

|  | **Intervention group (POC VL; n=411)** | **Control group (SOC VL; n=409)** | **Total (n=820)** |
| --- | --- | --- | --- |
| Live birth  Stillbirth  Miscarriage  Abortion  Neonatal death  Infant death  Maternal death  Missing | 357 (87.0%)  8 (2.0%)  1 (0.2%)  17 (4.1%)  6 (1.5%)  2 (0.5%)  1 (0.2%)  19 (4.6%) | 358 (87.5%)  7 (1.7%)  5 (1.2%)  16 (4.0%)  6 (1.5%)  0  1 (0.2%)  16 (4.0%) | 715 (87.2%)  15 (1.8%)  6 (0.7%)  33 (4.0%)  12 (1.5%)  2 (0.2%)  2 (0.2%)  35 (4.3%) |

**Supplemental Table 5: HIV testing and test results among infants born to Opt4Mamas participants, February 2019-August 2021**

|  | **Intervention group (POC VL; n=411)** | **Control group (SOC VL; n=409)** |
| --- | --- | --- |
| Number of infants known to be alive at:  Delivery  3 months postpartum  6 months postpartum  9  12  15  18 | 355 (99.4%)  349 (98.0%)  335 (94.0%)  33 (9.2%)  4 (1.2%)  0  0 | 356 (99.4%)  350 (98.0%)  345 (96.4%)  167 (46.6%)  104 (29.1%)  28 (8.0%)  7 (2.0%) |
| Number of infants alive and tested at:  Delivery  3 months postpartum  6 months postpartum  9  12  15  18 | 334 (81.3%)  329 (80.0%)  315 (76.6%)  28 (6.8%)  2 (0.5%)  0  0 | 319 (78.0%)  314 (76.8%)  312 (76.3%)  145 (35.5%)  88 (21.5%)  26 (6.4%)  7 (1.7%) |
| Test results by 6 months postpartum  HIV-infected  HIV-uninfected  Testing not done or recorded | 2 (0.5%)  339 (82.5%)  70 (17.0%) | 3 (0.7%)  326 (79.7%)  80 (19.5%) |
